# Diagnostic Performance of a Contrast-Enhanced Ultra-Low-Dose High-Pitch CT Protocol with Reduced Scan Range for Detection of Pulmonary Embolisms

**DOI:** 10.3390/diagnostics11071251

**Published:** 2021-07-13

**Authors:** Andreas S. Brendlin, Moritz T. Winkelmann, Felix Peisen, Christoph P. Artzner, Konstantin Nikolaou, Ahmed E. Othman, Saif Afat

**Affiliations:** 1 Department of Diagnostic and Interventional Radiology, Eberhard-Karls University, 72076 Tuebingen, Germany; andreas.brendlin@med.uni-tuebingen.de (A.S.B.); Moritz.Winkelmann@med.uni-tuebingen.de (M.T.W.); felix.peisen@med.uni-tuebingen.de (F.P.); christoph.artzner@med.uni-tuebingen.de (C.P.A.); konstantin.nikolaou@med.uni-tuebingen.de (K.N.); saif.afat@med.uni-tuebingen.de (S.A.); 2 Department of Neuroradiology, Johannes Gutenberg University, 55101 Mainz, Germany

**Keywords:** pulmonary embolism, emergency radiology, radiation dose reduction, iterative reconstruction, reduced scan range, 3rd generation dual-source CT

## Abstract

(1) Background: To evaluate the diagnostic performance of a simulated ultra-low-dose (ULD), high-pitch computed tomography pulmonary angiography (CTPA) protocol with low tube current (mAs) and reduced scan range for detection of pulmonary embolisms (PE). (2) Methods: We retrospectively included 130 consecutive patients (64 ± 16 years, 69 female) who underwent clinically indicated high-pitch CTPA examination for suspected acute PE on a 3rd generation dual-source CT scanner (SOMATOM FORCE, Siemens Healthineers, Forchheim, Germany). ULD datasets with a realistic simulation of 25% mAs, reduced scan range (aortic arch—basal pericardium), and Advanced Modeled Iterative Reconstruction (ADMIRE®, Siemens Healthineers, Forchheim, Germany) strength 5 were created. The effective radiation dose (ED) of both datasets (standard and ULD) was estimated using a dedicated dosimetry software solution. Subjective image quality and diagnostic confidence were evaluated independently by three reviewers using a 5-point Likert scale. Objective image quality was compared using noise measurements. For assessment of diagnostic accuracy, patients and pulmonary vessels were reviewed binarily for affection by PE, using standard CTPA protocol datasets as the reference standard. Percentual affection of pulmonary vessels by PE was computed for disease severity (modified Qanadli score). (3) Results: Mean ED in ULD protocol was 0.7 ± 0.3 mSv (16% of standard protocol: 4.3 ± 1.7 mSv, *p* < 0.001, *r* > 0.5). Comparing ULD to standard protocol, subjective image quality and diagnostic confidence were comparably good (*p* = 0.486, *r* > 0.5) and image noise was significantly lower in ULD (*p* < 0.001, *r* > 0.5). A total of 42 patients (32.2%) were affected by PE. ULD protocol had a segment-based false-negative rate of only 0.1%. Sensitivity for detection of any PE was 98.9% (95% CI, 97.2–99.7%), specificity was 100% (95% CI, 99.8–100%), and overall accuracy was 99.9% (95% CI, 98.6–100%). Diagnoses correlated strongly between ULD and standard protocol (Chi-square (1) = 42, *p* < 0.001) with a decrease in disease severity of only 0.48% (T = 1.667, *p* = 0.103). (4) Conclusions: Compared to a standard CTPA protocol, the proposed ULD protocol proved reliable in detecting and ruling out acute PE with good levels of image quality and diagnostic confidence, as well as significantly lower image noise, at 0.7 ± 0.3 mSv (84% dose reduction).

## 1. Introduction

Pulmonary artery embolisms (PE) are a common diagnosis and avertible cause of death in hospitalized patients [1]. Because of wide availability and the capability to quickly and reliably rule out this potentially deadly disease, computed tomography pulmonary angiography (CTPA) is the diagnostic method of choice [2]. While clinical capabilities have continually risen in the last decades, there has been an alarming rise in the incidence of PE as well [3]. The rising incidence of PE is indivisibly connected with an equally growing need for CTPA examinations, leading to increased patient exposure to radiation doses [4]. Multiple studies have voiced concern about this fact in the last decades, as its potentially harmful consequences are difficult to predict [5,6]. Managing radiation exposition according to the *As Low As Reasonably Achievable* (ALARA) principle has hence become a constant goal in clinical routine and radiological research. Radiation dose is, however, closely linked to image quality and image noise. Reducing radiation dose is, therefore, not an easy task, as it might severely limit diagnostic accuracy [7,8]. Solving this dilemma becomes especially important in younger patients and in pregnant women, where CTPA may result in elevated radiation exposure of the mammary glands [9,10]. In light of this background, there have been numerous approaches to reducing radiation doses in CT, particularly in CTPA. Apart from modulation of tube current (mAs) and reducing peak tube voltage (kVp), the introduction of the latest generation CT scanners and the resurgence of iterative reconstruction (IR) techniques have significantly contributed towards radiation exposure reductions in CTPA, enabling mean radiation doses below 2 mSv [11]. In addition, approaches using the reduction of the scan range in the z-axis have shown promising results in abdominopelvic CT and CTPA [12,13]. While these individual methods are proven to reduce radiation doses in CT significantly, to our knowledge, there have not been any attempts to evaluate the feasibility of a CTPA protocol that combines all of these approaches. Therefore, the scope of our study was to evaluate the effects of a retrospectively simulated ultra-low-dose (ULD) CTPA protocol at 25% radiation dose with reduced scan range, high-pitch scan mode, and iterative reconstruction techniques on diagnostic accuracy, image quality, and diagnostic confidence, as compared to a standard CTPA protocol.

## 2. Materials and Methods

### 2.1. Population

**Figure 1:**
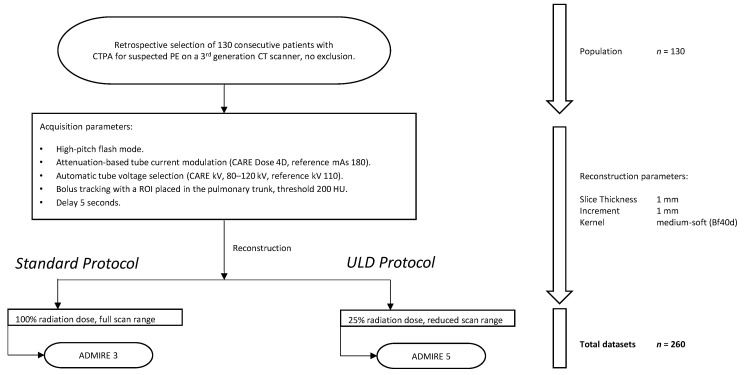
Patient enrollment and study workflow.

Retrospective collection of clinically indicated CTPA for suspected acute pulmonary embolisms for this study was approved by the institutional review board with a waiver for the need for informed consent. We included 130 consecutive patients (19–94 years, 69 female) who underwent clinically indicated CTPA for suspected PE from August to October 2019. No patients were excluded. To form a comprehensive image of our population, we collected biometric details such as sex, date of birth, the patients’ age, and BMI at the examination. Figure 1 is a flowchart of patient enrollment and the study workflow.

### 2.2. Image Acquisition and Reconstruction Parameters

All CT exams were acquired on the same 3rd generation dual-source CT Scanner (SOMATOM FORCE, Siemens Healthineers, Forchheim, Germany). For image acquisition, we used the proprietary single-energy high-pitch mode (“flash mode”) with attenuation-based tube current modulation (CARE Dose4D, reference mAs 180) and automatic tube voltage selection (80–120 kV, reference kV 110) matrix size set to 512 and field of view set to 50 cm. Collimation was 0.6 × 192 mm, pitch factor 2.8, and gantry rotation time 0.25 s. Depending on individual body weight, CTPA was automatically performed with 50–60 ml contrast medium (Imeron 400, Bracco, Milan, Italy), applied through an antecubital vein catheter. We used a dual syringe injector with a chaser of 40 ml saline, each at a flow rate of 3.5 mL/s (CT Stellant, Medrad, Indianola, PA, USA). Bolus tracking was used to ensure optimal contrast enhancement of the pulmonary arteries by placing a region of interest (ROI) with a trigger threshold of 200 Hounsfield units (HU) in the pulmonary trunk. Delay was 5 s. To avoid quality differences due to varying electronic noise levels, image reconstruction and low dose simulations were conducted solely using the proprietary reconstruction Software ReconCT (version 14.2.0.40998, Siemens Healthineers, Forchheim, Germany). We used a medium-hard kernel (Bv40d) with a slice thickness and increment of 1 mm in axial, coronal, and sagittal orientation. In accordance with our hospital’s standard CTPA protocol, we employed Advanced Modeled Iterative Reconstruction (ADMIRE^®^, Siemens Healthineers, Forchheim, Germany) strength3 for *standard protocol* reconstructions. For *ULD protocol* reconstructions, we performed a tube current simulation by reducing the mAs to 25% and employed a high level of iterative reconstruction (ADMIRE strength 5), as this combination showed promising results in other studies [11]. In the same step, we additionally simulated a limited scan range from the top of the aortic arch to the basal pericardium.

### 2.3. Radiation Dose

We collected the tube voltage (kV), the CT dose index (CTDI_vol_) and the dose length product (DLP) from the study protocol for every patient. To determine the effective radiation dose, we used a dedicated dosimetry software (Radimetrics ver. 2.9, Bayer Medical Care, Leverkusen, Germany) that also allows for the estimation of the effective radiation dose (ED) at 25% mAs, with a reduced scan range.

### 2.4. Analysis of Subjective Image Quality

Three radiologists (3–6 years experience in chest radiology) independently performed the subjective analysis of image quality blinded to the examinations’ clinical background information. All images were initially displayed in a soft tissue window (center 50 HU, width 350 HU), but individual adjustments were allowed for the reading. Assessment of subjective image quality and diagnostic confidence was performed according to the criteria listed in the chapter “Chest, general” of the European Guidelines on Quality Criteria in Computed Tomography [14], using an equidistant 5-point Likert scale (5 = very good, 4 = good, 3 = average, 2 = below average, 1 = poor).

### 2.5. Analysis of Objective Image Quality

We used image noise as a marker for objective image quality. We defined noise as the standard deviation of HU taken from homogenous regions of interest (ROI) with a size of ≥2 cm^2^ placed in the paraspinal muscles. Noise measurements were performed by each reviewer in the process of subjective image quality reading. We measured noise bilaterally in 5 consecutive slices and averaged all results for a mean overall noise value.

### 2.6. Diagnostic Accuracy and Disease Severity

**Figure 2:**
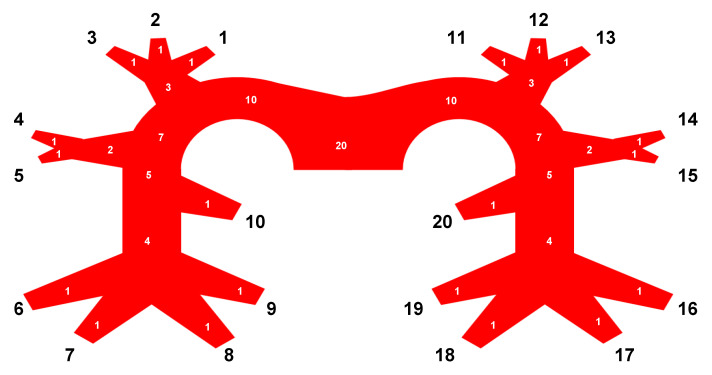
Pulmonary tree with vessel segments (black numbers) and modified Qanadli score points (white numbers) [15].

For diagnostic accuracy and assessment of disease severity, we used a modified Qanadli score [15]: each reader evaluated the images for the presence/absence of PE in the ten segmental vessels bilaterally and assigned a binary grade (0 = no PE, 1 = PE). Findings positive for PE ranged from partial intraluminal filling defects to complete arterial occlusions, proximal PE were counted as positive findings for all downstream segments. Figure 2 shows modified Qanadli score points (white numbers) for PE at the respective locations. Findings not covered by the given scan range in the *ULD protocol* were counted as false-negatives.

Vessel segments (black numbers) are defined in Table 1. Pulmonary lobes are abbreviated as follows: right upper lobe = RUL, right middle lobe = RML, right lower lobe = RLL, left upper lobe = LUL, left lower lobe = LLL.

We calculated the overall percentual affection of segmental vessels by PE as an indicator of severity. For reference standard, the original datasets were evaluated by a senior radiologist with eight years of experience in synopsis with the clinical reports.

### 2.7. Statistical Analysis

IBM^®^ SPSS^®^ Statistics Version 27 for Windows (Armonk, NY, USA) was used for statistical analysis. Data distribution was tested using the Shapiro–Wilk test. Normally distributed variables were expressed as mean ± standard deviation, non-normally distributed variables as median, and interquartile range (IQR). Normally distributed variables were analyzed using Student’s paired *t*-test, non-normally distributed variables with Wilcoxon’s paired *t*-test. A *p*-value ≤ 0.05 indicated statistical significance. We computed Pearson’s correlation (r) to measure effect size and defined r-values from 0.1 to 0.3 as indicative for a small, from 0.3 to 0.5 for a medium, and ≥0.5 for a large effect size. To measure inter-rater agreement, we used an intraclass correlation coefficient (ICC) [16]. ICC values of 0–0.2 were considered as slight, 0.21–0.4 as fair, 0.41–0.6 as moderate, 0.61–0.8 as substantial, and 0.81–1.00 as almost perfect levels of agreement. For patient-based diagnostic accuracy, we performed crosstabulations to calculate sensitivity and specificity with 95% confidence intervals. We computed the positive and negative likelihood ratio, positive and negative predictive value, as well as accuracy. We furthermore added a segment-based accuracy analysis via bootstrapping the pulmonary vessels to account for the clustered nature of the data. Pearson’s Chi-Square tests for the contingency of PE diagnosis on a vessel segment-based level ensued with Bonferroni correction to counteract Type I error increase in multiple comparisons. Cramer’s V was used as a symmetric measure with values > 0.3, indicating a strong correlation.

## 3. Results

### 3.1. Patient Population, Scan Range, and Radiation Dose

We included 130 patients (61 male, 69 female) in our study. Prevalence of PE was 32% (n = 42). A total of 88 patients (68%) showed other findings that were deemed causative for acute thoracic symptoms. Table 2 gives an overview of the patient characteristics and the radiation dose.

**Figure 3:**
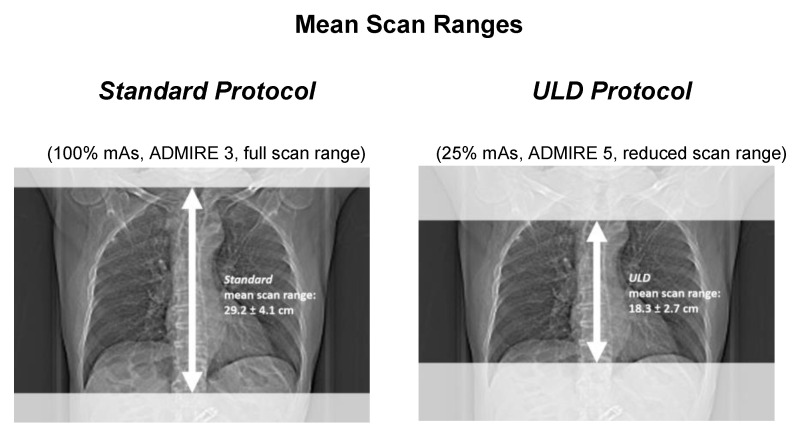
Visualization of scan lengths in full and reduced range protocol.

The mean ED of the standard protocol was 4.3 ± 1.7 mSv. For the ULD protocol, mean ED was 0.7 ± 0.3 mSv, resembling 16% of the initial radiation dose (*p* < 0.001). For visualization of scan ranges, see Figure 3.

The mean scan range of the standard protocol was 29.2 ± 4.1 cm. In the ULD protocol, the mean scan range was reduced to 18.3 ± 2.7 cm, which amounted to 63% of the original length.

### 3.2. Analysis of Subjective Image Quality

**Figure 4:**
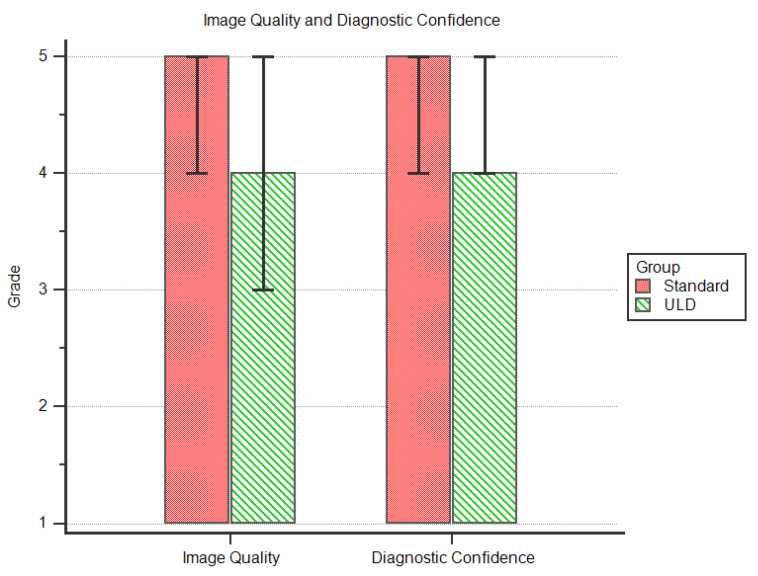
Comparison of image quality and diagnostic confidence.

Image quality was rated *very good* (5; IQR 4–5) for the *standard protocol* and *good* (4; IQR 3–5) for the *ULD protocol*. Diagnostic confidence was very good (5; IQR 4–5) in the *standard protocol* and *good* (4; IQR 4–5) for the *ULD protocol*. Figure 4 is a graph of the data of subjective image quality analysis.

There were no significant differences between the ratings of image quality (*p* = 0.486) and diagnostic confidence (*p* = 0.28) with effect size indicating a strong effect (*r* > 0.5) in both groups. For further details, see Table 3.

### 3.3. Analysis of Objective Image Quality

**Figure 5:**
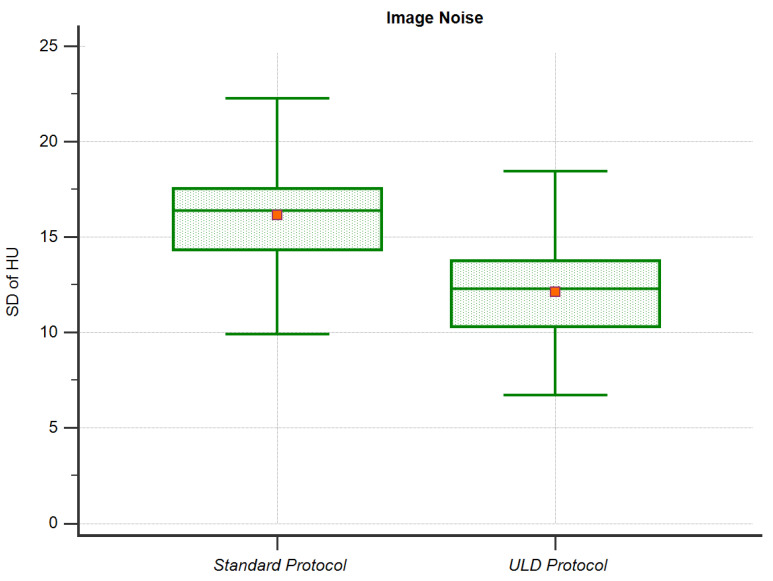
Image Noise.

For objective image quality, we analyzed image noise. Figure 5 is a graph of the comparison of image noise.

Image noise was 16.3 ± 2. 9 HU in the *standard protocol*. In the *ULD protocol*, noise was significantly lower (*p* < 0.001, *r* > 0.5) at 12.3 ± 3.0 HU.

### 3.4. Diagnostic Accuracy and Severity

#### 3.4.1. Patient-Based Accuracy

We had 42 cases of PE in our 130 patients, 39 of which were classified correctly in the *ULD protocol*. Scan range reduction led to 3 false-negative findings and 0 false-positive classification of PE by all three readers (ICC = 1). Patient-based sensitivity of the ULD protocol was 92.9% (95% CI, 80.5–98.5%) and Specificity 100% (95% CI, 95.9–100%). The positive predictive value was 100%, the negative predictive value 96.7% (95% CI, 90.8–99.9%). The overall accuracy (probability that a patient is classified correctly) of the ULD protocol was 97.7% (95% CI, 93.4–99.5%). See Table 4 for further details.

#### 3.4.2. Segment-Based Accuracy

In our population, 359 out of 2600 segmental vessels were affected by PE (13.7% prevalence). Of those, a total of 355 vessels were classified correctly in the ULD protocol (98.9%). Scan range reduction led to four false-negative findings (0.1%) and no false-positive classifications of PE by all three readers (ICC = 1). Segment-based sensitivity in the ULD protocol was 98.9% (95% CI 97.2–99.7%) and specificity 100% (95% CI, 99.8–100%). As in patient-based analysis, positive predictive value was 100%. The negative predictive value 99.8% (95% CI, 97.5–99.9%). The ULD protocol allowed for overall segment-based accuracy (probability that a segment is correctly classified as affected by PE) of 99.9% (95% CI, 98.6–100%). See Table 5 for further details.

Chi-square tests with Bonferroni-corrected significance levels showed a strong correlation between the segment-based classifications of PE in *standard protocol* and *ULD protocol* (Chi-square (1) = 42, *p* < 0.001, Fisher’s exact test *p* < 0.001; Cramer’s V ≥ 0.906, *p* < 0.001). See Appendix A, Table A1 for further details.

#### 3.4.3. Disease Severity

**Figure 6:**
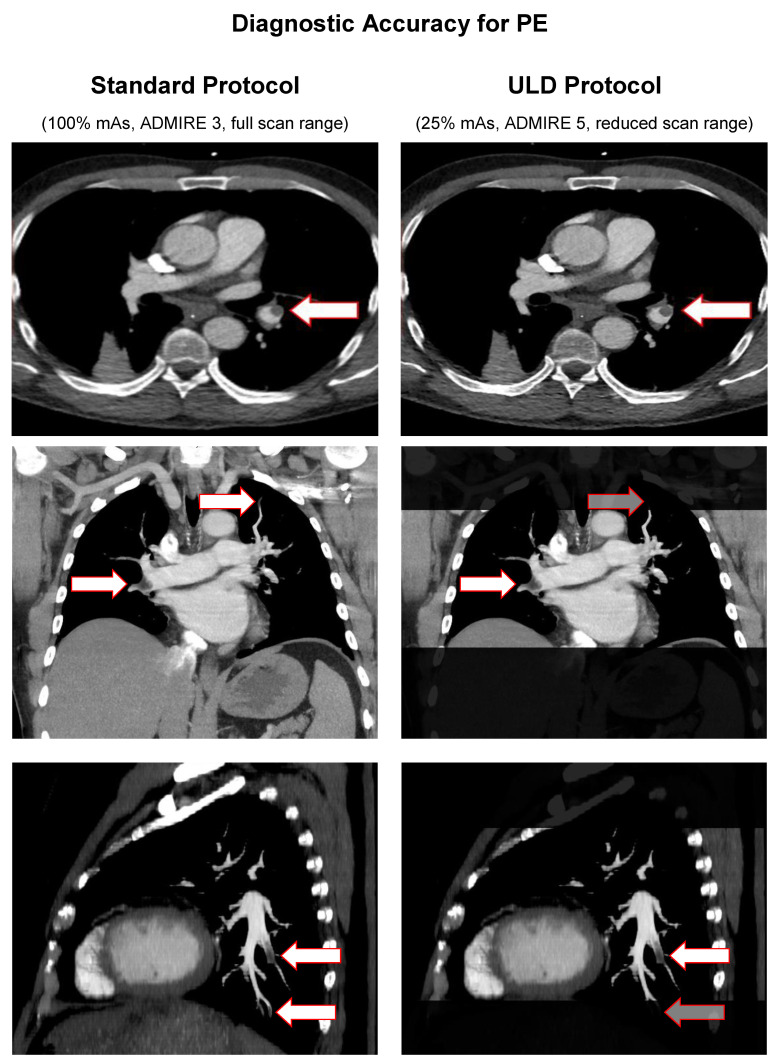
Diagnostic accuracy for PE in standard protocol vs. ULD protocol.

With a maximum of 20 awardable points, *standard protocol* yielded a mean modified Qanadli score of 8.55 ± 6.271 points vs. *ULD protocol* with 8.45 ± 6.283 points. Mean severity (the percentage of affected vessels) was 42.74% ± 31.356 in *standard protocol* and 42.26% ± 31.434 in *ULD protocol*. Pearson’s correlation showed a strong coherency (*r* = 0.998, *p* < 0.001) between the modified Qanadli score measurements, as well as severity in both protocols. Student’s *t*-test for paired samples showed no significant differences (T = 1.667, *p* = 0.103) between the modified Qanadli score measurements and consecutively the severity in both protocols. Figure 6 illustrates diagnostic accuracy for PE in our population with example images from three patients for both protocols. Greyed out arrows mark false-negative findings.

## 4. Discussion

This study aimed to evaluate the diagnostic performance of a contrast-enhanced ULD high-pitch CTPA protocol with a reduced scan range for detection of pulmonary embolisms. We included 130 consecutive patients with CTPA flash mode scans at a 3rd generation CT scanner. For the *standard protocol* (100% mAs, ADMIRE 3, full scan range), the mean effective radiation dose was 4.3 ± 1.7 mSv compared to 0.7 ± 0.3 mSv in the *ULD protocol* (25% mAs, ADMIRE 5, reduced scan range), a decrease of 84% (*p* < 0.001, *r* > 0.5).

The possibilities of radiation dose reduction in CTPA for suspected PE have been explored in some studies before. Concerning mAs reduction, Sauter et al. reported excellent levels of diagnostic accuracy of PE in combination with iterative reconstruction at effective radiation doses as low as 0.9 mSv [17]. For scan range reduction, Atalay et al. identified a possible decrease to 53% coverage in their distribution-based study regarding scan length optimization at equal levels of diagnostic accuracy [13]. If applied to our dataset, this method would result in a radiation dose target of 2.3 mSv. The proposed *ULD protocol* combines these approaches, yielding the possibility of a tremendous reduction in effective radiation dose down to 16% (0.7 ± 0.3 mSv).

Image quality and diagnostic confidence were *good* in the proposed *ULD protocol* without any significant differences compared to the ratings of the *standard protocol*. These results are accordant to multiple studies that have shown high levels of subjective image quality and diagnostic confidence when using low dose approaches combined with iterative reconstruction algorithms [17,18,19]. In agreement with our study, Sauter et al. reported good levels of subjective image quality and diagnostic confidence down to 25% radiation dose with a target of 0.9 mSv [17]. At 0.7 ± 0.3 mSv, the proposed protocol is capable of reducing radiation dose even further. We found image noise to be significantly lower in the proposed *ULD protocol* than in the *standard protocol*., a fact most certainly due to the higher iterative reconstruction strength used for the ULD protocol. This is following previous results that have reported a continuous decrease of image noise with higher levels of model-based iterative reconstruction, ADMIRE in particular [20].

At a disease prevalence of 32.2%, the proposed *ULD protocol* yielded excellent values for sensitivity, specificity, and overall accuracy for ruling out pulmonary embolisms on a patient-based level, as well as on a segment-based level. We further measured a strong consistency between the segment-based classifications of PE in both protocols, attesting to the high reliability of the proposed *ULD protocol*. Furthermore, there was a strong coherency between the measurements of the modified Qanadli score and the severity in both protocols. Regarding diagnostic accuracy of PE in combination with scan range reduction, multiple studies have shown only minimal distribution of PE in the apical vessel segments above the aortic arch or the lower segments below the base of the heart [21,22]. This is particularly interesting, as there is still discussion about the clinical significance of such emboli, especially if weighed against the individual risks of anticoagulation [23,24,25,26,27]. Although the modified Qanadli score and the severity measurements were expectedly lower in the proposed *ULD protocol*, the decrease of 0.1 points in Qanadli score and 0.48% in severity weren’t statistically significant. Therefore, this protocol may be beneficial for patients by reducing unnecessary radiation exposure.

## 5. Limitations

Our study has several limitations. Firstly, we chose a retrospective study design with simulated radiation dose reduction to minimize unwanted radiation dose application, whereas a prospective study design might have further limited biases and might have allowed to draw conclusions on clinical decision-making. Secondly, this study only investigated the diagnostic performance and accuracy of the *ULD protocol* regarding PE. As we had several patients with other, or even no findings that were causative of acute thoracic symptoms, a systematic review of the proposed *ULD protocol* concerning such cases, therefore, needs to remain the scope of future studies. Thirdly, we need to mention that this study was performed using a high-end 3rd generation CT scanner (SOMATOM Force) with iterative reconstructions, which is not available at every clinical site. Our results might, therefore, not necessarily be transformable to other setups, or older scanner generations.

## 6. Conclusions

Compared to a standard CTPA protocol, the proposed ULD protocol proves reliable in detecting and ruling out acute PE with good levels of image quality and diagnostic confidence, as well as significantly lower image noise, at 0.7 ± 0.3 mSv (84% dose reduction).

## Figures and Tables

**Table 1:** Definitions of pulmonary vessel segments.

#	Lobe	Vessel Segment	#	Lobe	Vessel Segment
1	RUL	Anterior segment	11	LUL	Anterior segment
2	RUL	Apical segment	12	LUL	Anterior apical segment
3	RUL	Posterior segment	13	LUL	Apical posterior segment
4	RML	Apical segment	14	LUL	Apical lingula segment
5	RML	Basal segment	15	LUL	Basal lingula segment
6	RLL	Lateral basal segment	16	LLL	Lateral segment
7	RLL	Anterior basal segment	17	LLL	Anteromedial segment
8	RLL	Medial basal segment	18	LLL	Posteromedial segment
9	RLL	Posterior basal segment	19	LLL	Posterolateral segment
10	RLL	Superior segment	20	LLL	Superior segment

**Table 2:** Patient characteristics and radiation dose.

Parameter	Male	Female	Total
Patient population			
Absolute (n)	61	69	130
Reconstructions (n)	122	138	260
Mean age (y)	65 ± 16	63 ± 18	64 ± 16
Mean BMI	27.7 ± 2.4	27.6 ± 1.7	27.7 ± 2.1
Contrast medium (mL)	62 ± 21	54 ± 20	58 ± 20
Scan range			
Mean scan range in standard protocol (cm)	29.7 ± 4.1	28.7 ± 4.1	29.2 ± 4.1
Mean scan range in ULD protocol (cm)	18.7 ± 2.7	17.9 ± 2.7	18.3 ± 2.7
Diagnosis (n)			
Pulmonary embolism	20	22	42
Other findings causative of acute thoracic symptoms			
Pleural effusion	22	24	46
Pulmonary xongestion	14	9	23
Pulmonary infiltrate	7	3	10
Pneumothorax	4	2	6
No causative findings	1	2	3
kV	108 (80–120)	104 (80–120)	106 (80–120)
mAs	197	190	193
mAs ref	180	180	180
CTDI	6.93 ± 2.76	7.07 ± 3.5	7 ± 3.17
DLP (mGy)	243.4 ± 96.31	236.26 ± 91.86	239.61 ± 95.82
Mean effective radiation dose (ED)			
*Standard protocol* (100% mAs, ADMIRE 3, full scan range)	4.0 ± 1.6	4.6 ± 1.8	4.3 ± 1.7
*ULD protocol* (25% mAs, ADMIRE 5, reduced scan range)	0.7 ± 0.3	0.8 ± 0.3	0.7 ± 0.3

**Table 3:** Ratings of image quality and diagnostic confidence.

	Protocol	ED	Rating			ICC	ICC: 95% CI			*Standard* vs. *ULD*	
		(mSv)	Median	IQR		Av. Measure	Lower Bound		Upper Bound	*p*	*r*
Image quality	*Standard*	4.3 ± 1.7	5	4	5	0.971	0.961	0.979		0.486	>0.5
	*ULD*	0.7 ± 0.3	4	3	5	0.967	0.956	0.976			
Diagnostic Confidence	*Standard*	4.3 ± 1.7	5	4	5	0.979	0.955	0.975		0.28	>0.5
	*ULD*	0.7 ± 0.3	4	4	5	0.953	0.937	0.965			

**Table 4:** ULD—Patient-Based Sensitivity and Specificity.

Protocol	ED		Value	ICC: 95% CI	
	(mSv)	Patient-Based Analysis		Lower Bound	Upper Bound
*ULD*	0.7 ± 0.3	Sensitivity	92.9%	80.5	98.5
		Specificity	100%	95.9	100
		Positive likelihood ratio			
		Negative likelihood ratio	0.1	0	0.2
		Disease prevalence	32.3%	24.4	41.1
		Positive predictive value	100%		
		Negative predictive value	96.7%	90.8	98.9
		Accuracy	97.7%	93.4	99.5

**Table 5:** ULD—segment-based sensitivity and specificity.

Protocol	ED		Value	ICC: 95% CI	
	(mSv)	Segment-Based Analysis		Lower Bound	Upper Bound
*ULD*	0.7 ± 0.3	Sensitivity	98.9%	97.2	99.7
		Specificity	100%	99.8	100
		Positive likelihood ratio			
		Negative likelihood ratio	0.01	0	0.3
		Disease prevalence	13.81%	12.5	15.2
		Positive predictive value	100%		
		Negative predictive value	99.8%	99.5	99.9
		Accuracy	99.9%	98.6	100

## Data Availability

Data sharing not applicable.

## Appendix A

**Table A1:** Segment-based crosstabulation, Chi-square tests, and symmetric measures.

Standard Protocol * ULD Protocol Crosstabulation									Chi-Square Tests				Symmetric Measures	
Segment			Count			% within Standard								
			ULD		Total	ULD		Total	Pearson Chi-Square			Fisher’s Exact Test	Nominal Cramer’s V	
			1	0		1	0		Value	df	Asymptotic Significance (2-sided)	Exact Significance (2-sided)	Approximate Significance	Value
01 RUL anterior segment	Standard	1	20	0	20	100.0%	0.0%	100.0%	42.000	1	0.000	0.000	0.000	1.000
		0	0	22	22	0.0%	100.0%	100.0%						
02 RUL apical segment	Standard	1	20	0	20	100.0%	0.0%	100.0%	42.000	1	0.000	0.000	0.000	1.000
		0	0	22	22	0.0%	100.0%	100.0%						
03 RUL posterior segment	Standard	1	19	0	19	100.0%	0.0%	100.0%	42.000	1	0.000	0.000	0.000	1.000
		0	0	23	23	0.0%	100.0%	100.0%						
04 RML apical segment	Standard	1	22	0	22	100.0%	0.0%	100.0%	42.000	1	0.000	0.000	0.000	1.000
		0	0	20	20	0.0%	100.0%	100.0%						
05 RML basal segment	Standard	1	22	0	22	100.0%	0.0%	100.0%	42.000	1	0.000	0.000	0.000	1.000
		0	0	20	20	0.0%	100.0%	100.0%						
06 RLL lateral basal segment	Standard	1	21	0	21	100.0%	0.0%	100.0%	42.000	1	0.000	0.000	0.000	1.000
		0	0	21	21	0.0%	100.0%	100.0%						
07 RLL anterior basal segment	Standard	1	18	0	18	100.0%	0.0%	100.0%	42.000	1	0.000	0.000	0.000	1.000
		0	0	24	24	0.0%	100.0%	100.0%						
08 RLL medial basal segment	Standard	1	20	0	20	100.0%	0.0%	100.0%	42.000	1	0.000	0.000	0.000	1.000
		0	0	22	22	0.0%	100.0%	100.0%						
09 RLL posterior basal segment	Standard	1	26	0	26	100.0%	0.0%	100.0%	42.000	1	0.000	0.000	0.000	1.000
		0	0	16	16	0.0%	100.0%	100.0%						
10 RLL superior segment	Standard	1	19	0	19	100.0%	0.0%	100.0%	42.000	1	0.000	0.000	0.000	1.000
		0	0	23	23	0.0%	100.0%	100.0%						
11 LUL anterior segment	Standard	1	13	0	13	100.0%	0.0%	100.0%	42.000	1	0.000	0.000	0.000	1.000
		0	0	29	29	0.0%	100.0%	100.0%						
12 LUL anterior apical segment	Standard	1	13	0	13	100.0%	0.0%	100.0%	42.000	1	0.000	0.000	0.000	1.000
		0	0	29	29	0.0%	100.0%	100.0%						
13 LUL apical posterior segment	Standard	1	16	2	18	88.9%	11.1%	100.0%	34.462	1	0.000	0.000	0.000	0.906
		0	0	24	24	0.0%	100.0%	100.0%						(95% CI 0.751–1.000)
14 LUL apical lingula segment	Standard	1	12	0	12	100.0%	0.0%	100.0%	42.000	1	0.000	0.000	0.000	1.000
		0	0	30	30	0.0%	100.0%	100.0%						
15 LUL basal lingula segment	Standard	1	12	0	12	100.0%	0.0%	100.0%	42.000	1	0.000	0.000	0.000	1.000
		0	0	30	30	0.0%	100.0%	100.0%						
16 LLL lateral segment	Standard	1	16	0	16	100.0%	0.0%	100.0%	42.000	1	0.000	0.000	0.000	1.000
		0	0	26	26	0.0%	100.0%	100.0%						
17 LLL anteromedial segment	Standard	1	15	0	15	100.0%	0.0%	100.0%	42.000	1	0.000	0.000	0.000	1.000
		0	0	27	27	0.0%	100.0%	100.0%						
18 LLL posteromedial segment	Standard	1	21	1	22	95.5%	4.5%	100.0%	38.182	1	0.000	0.000	0.000	0.953
		0	0	20	20	0.0%	100.0%	100.0%						(95% CI 0.904–1.000)
19 LLL posterolateral segment	Standard	1	21	1	22	95.5%	4.5%	100.0%	38.182	1	0.000	0.000	0.000	0.953
		0	0	20	20	0.0%	100.0%	100.0%						(95% CI 0.898–1.000)
20 LLL superior segment	Standard	1	9	0	9	100.0%	0.0%	100.0%	42.000	1	0.000	0.000	0.000	1.000
		0	0	33	33	0.0%	100.0%	100.0%						
